# A community-based randomized controlled trial providing weekly iron-folic acid supplementation increased serum- ferritin, -folate and hemoglobin concentration of adolescent girls in southern Ethiopia

**DOI:** 10.1038/s41598-021-89115-5

**Published:** 2021-05-06

**Authors:** Yoseph Halala Handiso, Tefera Belachew, Cherinet Abuye, Abdulhalik Workicho, Kaleab Baye

**Affiliations:** 1 School of Public Health, College of Health Sciences and Medicine, Wolaita Sodo University, Wolaita Sodo, Ethiopia; 2 Department of Nutrition and Dietetics, Faculty of Public Health, Jimma University, Jimma, Ethiopia; 3 ENGINE/Save the Children, Addis Ababa, Ethiopia; 4 Friedman School of Nutrition Science and Policy, Tufts University, Boston, MA USA; 5 Center for Food Science and Nutrition, College of Natural and Computational Sciences, Addis Ababa University, Addis Ababa, Ethiopia

**Keywords:** Epidemiology, Nutrition

## Abstract

Adequate micronutrient status during adolescence can break the inter-generational cycle of malnutrition. This study evaluated the effect of community-based weekly iron-folic acid supplementation (WIFAS) on serum ferritin (SF), serum folate (SFol) and hemoglobin concentration (Hb) among adolescent girls. A community-based, individually randomized-controlled trial (RCT) was conducted in four villages of Wolaita and Hadiya zones. Adolescent girls (n = 226) aged 10–19 years were recruited and randomly assigned (n = 113/group) into: (i) WIFAS and (ii) control (no intervention) groups. Anthropometry, Hb concentration, and serum ferritin (SF), SFol, and C-reactive protein (CRP) was analyzed at baseline and endline. Baseline Hb, SF, SFol and CRP concentrations were similar in both groups (P > 0.05). About 47–49% of adolescents had marginal iron store (< 50 µg/l). Hb, SF, and SFol concentrations increased in the intervention group, but not in the control group (P < 0.05). Marginal iron store decreased from 49 to 12% after 3-months of WIFAS; whereas, the proportion of adolescents with elevated SF (> 15 µg/l) was slightly higher in the WIFAS than in the control group (P = 0.06). After adjusting for confounding factors in the multiple linear regression model, a three-months WIFAS intervention was associated with an improvement of 4.10 ng/ml in serum folate, 39.1 μg/l in serum ferritin, and 1.2 g/dl in hemoglobin concentration relative to the control group (P < 0.001). WIFAS intervention for three-months was effective in reducing iron and folate deficiency in adolescent girls. Future studies should evaluate the long-term impact of intermittent WIFAS.

## Introduction

Anemia is widely prevalent in low and middle-income countries (LMIC) and disproportionately affects women and children^1^. Anemia has been linked to adverse outcomes including poor cognitive and physical performance, increased susceptibility to disease, and perinatal complications like low-birth weight, still-birth, and preterm birth^2,3^. The etiology of anemia is complex and can be of nutritional origin or related to infection or chronic diseases^4^. Among nutritional causes, iron and folic acid deficiencies are the most common in women and relate to the higher physiological needs to compensate for menstrual losses and support a healthy pregnancy and fetal development^5,6^. Consequently, iron-folic acid (IFA) interventions have been the mainstay of anemia prevention interventions^7^.

IFA supplementation has for a long time been considered primarily for pregnant women. This is considering the increased physiological iron and folic acid requirement of the mother and the added demand to support the development of the fetus, but also to prevent neural tube defects^8,9^. However, in LMICs like Ethiopia, women attend their first antenatal care (ANC) visit quite late in their first trimester or often in their second trimester. Besides, adherence to recommendations is low and is often complicated by inadequate supply, social norms, and side effects^10–14^. Altogether, the late and suboptimal adherence of IFA supplementation suggests that it may be strategic to increase the iron stores of adolescent girls and non-pregnant women of reproductive age through weekly IFA supplementation^15,16^. This will allow women to start pregnancy with adequate iron stores and folate status; hence, lowering the risk of iron-deficiency anemia and folate deficiencies. Weekly IFA supplementation can also have the added advantage of supporting cognitive-^17^ and the physical-performance of adolescents^18^.

The focus on adolescence as a window of opportunity for preventive nutrition interventions is quite recent^19^. Consequently, very few studies have evaluated preventive nutrition interventions including weekly IFA supplementation, and the quality of the evidence remains low as recently evaluated by a systematic review^20^. Recent studies from India, Nepal, and Kenya have all shown positive effects of weekly IFA supplementation on adolescents’ hemoglobin concentration^16,21,22^. These studies were all school-based and hence did not capture out of school girls that may be more at risk. They also did not measure markers of iron, folate, and inflammation status. Consequently, studies on the impact of community-based weekly IFA supplementation on anemia and iron and folate status of adolescents are urgently needed. Therefore, the present study aimed to investigate the effect of community-based weekly supplementation of IFA for three-months on serum ferritin, folate and hemoglobin level among adolescent girls in Wolaita and Hadiya zones, southern Ethiopia. We hypothesized that weekly Iron-folic acid supplementation improves serum ferritin, folate, and hemoglobin concentrations.

## Results

From the enrolled participants, 81% from the intervention and 99% from the control group completed the study. Nineteen adolescents discontinued the supplementation due to potential side-effects as they have reported vomit, nausea and constipation. Another two, discontinued because they left the villages where the supplementation was taking place. Study subjects were on average 14 years of age and came from an average household size of seven. Close to 80% were in high-school (grades 9–12), and about 4% had no formal education (Table 1).

**Table 1:** Socio-demographic characteristics of the adolescent girls.

	Level	WIFAS (n = 92)	Control (n = 112)	P-value
		n (%) or mean ± SD	n (%) or mean ± SD	
Age	10–13	32 (34.8)	39 (34.8)	
	14–16	56 (60.9)	64 (57.1)	0.18
	17–19	4 (4.3)	9 (8.0)	
Educational status	No formal education	4 (4.3)	5 (4.5)	
	grade 1–8	25 (27.2)	20 (17.9)	0.18
	grade 9–12	51 (55.4)	67 (59.8)	
	College/university	12 (13.0)	20 (17.9)	
Family size	< 5	16 (17.4)	26 (23.2)	0.20
	> 5	76 (82.6)	86 (76.8)	
Weight (kg)		43.16 ± 8.7	41.8 ± 8.1	0.26
MUAC		21.97 ± 2.13	20.64 ± 2.06	0.79

*MUAC*, mid-upper arm circumference, *WIFAS* weekly iron-folic acid supplementation.

There was no statistically significant difference in the mean hemoglobin, serum ferritin, serum folate, and CRP concentrations (P > 0.05), suggesting effective randomization (Table 2). Similarly, the proportion of subjects with depleted iron stores (serum ferritin < 15 µg/l), marginal and excess iron store were not statistically different between the two groups. The proportion of subjects with depleted and the excess iron store was low (≤ 5%), but close to half of the subjects had marginal iron stores. About 10% in the treatment and 13% in the control group had folate deficiency (P > 0.05).

**Table 2:** Hemoglobin, iron, and folate status of adolescent girls at baseline.

	WIFAS (n = 92)	Control (n = 112)	P-value
	Mean ± SD or %		
Hemoglobin (g/dl)	13.3 ± 1.4	13.1 ± 0.7	0.15
Serum ferritin (µg/l)	58.5 ± 35.2	61.1 ± 39.1	0.92
Serum folate (ng/ml)	10.2 ± 4.3	11.2 ± 4.6	0.33
CRP (µg/l)	0.66 ± 1.3	0.56 ± 1.2	0.63
Low ferritin level (< 15 µg/l)	5%	3%	0.08
Marginal iron store (< 50 µg/l)	45%	53%	0.97
Excess iron store (> 150 µg/l)	3%	4%	0.32
Folate deficiency (< 6 ng/ml)	10%	13%	0.72

*CRP* C-reactive protein, *SD* standard division, *WIFAS* weekly iron-folic acid supplementation; means were compared using t-test.

P-values < 0.05 were considered statistically significant.

Significant differences in hemoglobin, serum ferritin, and serum folate concentrations were observed between the intervention and the control group after 3 months of supplementation (Table 3), while no significant changes were observed in the control group. At endline, no adolescent in the intervention group had a depleted iron store or were folate deficient. The proportion of adolescents with marginal iron stores decreased from 45% at baseline to 11% at endline. During the same period, excess iron stores, reflected by a serum ferritin > 150 µg/l, increased from 3 to 10%.

**Table 3:** Hemoglobin, iron, and folate status of adolescent girls at endline.

	WIFAS (n = 92)	Control (n = 112)	P-value
	Mean ± SD		
Hemoglobin (g/dl)	14.5 ± 1.3	13.2 ± 0.7	< 0.001
Serum ferritin (µg/l)	97.8 ± 68.2	63.1 ± 45.9	< 0.001
Serum folate (ng/ml)	14.3 ± 4.6	11.4 ± 4.3	< 0.001
CRP (µg/l)	1.2 ± 3.0	0.82 ± 1.8	0.353
Low ferritin level (< 15 µg/l)	0%	4%	0.045
Marginal iron store (< 50 µg/l)	11%	54%	< 0.001
Excess iron store (> 150 µg/l)	10%	4%	0.066
Folate deficiency (< 6 ng/ml)	0%	11%	< 0.001

*SD* standard division, *WIFAS* weekly iron-folic acid supplementation; means were compared using t-test.

P-values < 0.05 were considered statistically significant.

The multivariate linear regression showed significant effects of the WIFAS program on endline serum folate, serum ferritin, and hemoglobin concentrations (Table 4). The lower the baseline ferritin, folate and hemoglobin concentrations, the higher the impact of the WIFAS program (P ≤ 0.001). Three months WIFAS intervention was associated with an improvement of 4.10 ng/ml in serum folate, 39.1 μg/l, and 1.2 g/dl in hemoglobin concentration relative to the control group (P < 0.001).

**Table 4:** Multiple linear regression for the association between WIFAS and serum ferritin, serum folate and hemoglobin concentration.

	Serum folate		Serum ferritin		Hemoglobin concentration	
	β [95.0%CI]	P-value	β [95.0%CI]	P-value	β [95.0%CI]	P-value
IFA supplementation	4.10 [1.841; 4.590]	< 0.001	39.076 [12.329; 47.822]	< 0.001	1.200[ 0.853; 1.629]	< 0.001
Age	− 0.013 [− 0.047; 0.021]	0.459	− 0.081 [− 0.533; 0.371]	0.725	0.004 [− 0.013; 0.006]	0.424
Family size	− 0.022 [− 0.349; 0.305]	0.894	0.138 [− 4.291; 4.567]	0.951	0.009 [− 0.083; 0.101]	0.847
Height	− 0.018 [− 0.119; 0.083]	0.731	− 0.357 [− 1.717; 1.003]	0.605	− 0.008 [− 0.036; 0.021]	0.583
Weight	0.117 [− 0.028; 0.262]	0.114	0.177 [− 1.803; 2.157]	0.860	− 0.006 [− 0.047; 0.035]	0.761
MUAC	− 0.266 [− 0.783; 0.251]	0.311	1.427 [− 5.555; 8.410]	0.687	0.057 [− 0.089; 0.203]	0.445
Baseline hemoglobin	− 0.009 [− 0.516; 0.497]	0.971	4.594 [− 2.229; 11.417]	0.186	− 0.800 [− 0.943; − 0.657]	< 0.001
Baseline CRP	0.229 [− 0.251; 0.709]	0.347	− 0.413 [− 6.896; 6.070]	0.900	− 0.009 [− 0.144; 0.127]	0.900
Baseline serum ferritin	− 0.001 [− 0.017; 0.014]	0.859	− 0.382 [− 0.596; − 0.168]	< 0.001	0.002 [− 0.002; 0.006]	0.379
Baseline serum folate	− 0.499 [− 0.628; − 0.370]	< 0.001	0.276 [− 1.476; 2.027]	0.757	0.011[− 0.026; 0.047]	0.561

Results are from multiple linear regression; ß = Unstandardized Coefficients; The maximum variance inflation factor (VIF) for serum ferritin status was 6.045, 5.984 for hemoglobin concentration and serum folate, respectively.

## Discussion

We investigated the effect of community-based WIFAS intervention among adolescent girls in southern Ethiopia. To our knowledge, this is the first study to examine the effect of community-based WIFAS on hemoglobin concentration, serum ferritin and serum folate concentrations of adolescent girls in southern Ethiopia. The prevalence of low serum ferritin was surprisingly low (< 5%), but marginal iron store was highly prevalent in about half of the studied adolescents. WIFAS significantly improved hemoglobin, folate and ferritin concentrations. Baseline hemoglobin, ferritin, and folate concentration was inversely associated with endline values.

The present study showed that iron deficiency was low, despite a predominantly cereal-based diet and the low availability of iron-fortified foods, as reported elsewhere^23^. This finding is in line with a recent study from Hirna, Ethiopia^24^. Both the present and the earlier study from Hirna identified high levels of marginal iron stores. Such low iron stores can increase the risk of iron deficiency in the event of increased demands due to pregnancy or excessive blood loss. This along with folate deficiency in about 10% of the adolescents and the positive response in serum folate and ferritin in the WIFAS intervention group suggests that the intervention is adapted to the context^22,25–27^. This is further confirmed by the higher response to the intervention in those with lower serum ferritin, folate and hemoglobin at baseline.

The elimination of folate deficiency after 3 months of WIFAS further highlights the importance of WIFAS intervention. Folate deficiency in the first 2 to 3 weeks has been linked to increased risks to congenital malformations^28–30^. Although folic acid supplementation is promoted during pregnancy, the delayed first antenatal care means that most pregnant women do not get folic acid supplements at the time when they need it the most (i.e. first 2 weeks of conception). In contrast, the WIFAS program allows adolescents to have an adequate folate status; hence, reducing the risk of deficiency, if and when they become pregnant^29,31^. The weekly regimen is also likely to decrease any of the possible side-effects observed during daily supplementation^21^.

The WIFAS program has been promoted in various LMIC through school-systems^25,32^. Many impact evaluations have shown the benefits of such platforms for constant monitoring and reducing school dropouts. Despite the advantages that the school system provides, in LMICs like Ethiopia, a high number of adolescents are out of school and thus cannot benefit from such programs^33^. Unless systems’ are in place to reach out of school adolescent girls, the WIFAS intervention for those going to school only is likely to widen inequalities between adolescent girls. Our study provides evidence that a community-based WIFAS program can be successful in improving serum micronutrient status to improvement levels observed in more controlled school settings. However, the rise in high serum ferritin suggests that there is a potential risk of iron overload for a segment of the adolescent population. Whenever possible, assessing iron status prior to WIFAS interventions can minimize possible adverse effects.

The present study has several strengths and limitations. Key strengths include the use of a randomized controlled trial and the implementation of a community-based WIFAS program. The measurement of inflammation marker using CRP measures is also an additional strength that increases the reliability of serum-ferritin and -folate measures. Measurement of α-glycoprotein (AGP), in addition to CRP, could have allowed us to adjust the serum ferritin values, instead of excluding subjects with inflammation. Nevertheless, samples with CRP > 5 mg/l were excluded from the analyses. The excluded subjects represented 2.5% of the total sample, were balanced by intervention group (P > 0.05), and had similar socio-demographic characteristics than subjects retained in the analyses. The study was an effectiveness study; hence, although we know that adherence to the WIFAS was generally high; the compliance to the regimen was not strictly monitored. The long-term impact of the WIFAS benefits remains unknown and warrants to be studied.

Notwithstanding the above limitations, the present study highlighted the beneficial impact of a community-based WIFAS program. The community-based WIFAS program significantly improved serum ferritin, serum folate, and blood hemoglobin concentration among adolescent girls proving this concept in the context of Ethiopia. The present finding suggests the potential benefit of scaling-up community-based WIFAS programs in Ethiopia and beyond. Future studies should evaluate the long-term benefits of such programs.

## Methods and subjects

### Study area and study design

A community-based, individually randomized trial (RCT) was conducted at four Villages of Wolaita and Hadiya zones using a two-arm parallel design (one intervention group and one control group). Wolaita and Hadiya zones are two zonal administrations in Southern Nations, Nationalities, and Peoples’ Region (SNNPR). These zones are predominantly dependent on agriculture, practice mixed crop-livestock production and live in permanent settlements. Within their landholdings, community members cultivate fruits, vegetables, roots, tuber crops, and cereals. Participants were randomly assigned into intervention or control group. The intervention group took weekly IFA supplementation, and the control group received nothing. The IFA supplement contained 60 mg of elemental iron and 0.4 mg of folic acid. The IFA supplementation was monitored by four clinical nurses and two supervisors recruited for the study. IFA tablets were provided every weekend through household visits. These household visits also provided a platform to answer any questions by the study subjects. The study period was from April to September 2019. The baseline survey was conducted in April–May, followed by a three-month supplementation period (June–August), and an endline survey in September.

### Ethical considerations

The study was approved by the institutional review board (IRB) of Wolaita Sodo University. All methods were performed in accordance to the Helsinki Declaration ethical principles for medical research involving human subjects. Official letter of cooperation was written to Wolaita and Hadiya zones, and the district health office. The nature of the study was fully explained to the study participants and their parents. Informed verbal and written consent/assent were obtained from parents/respondents before the interview and the intervention. The collected data were kept confidential. The trial is registered at the PanAfrican clinical trial registry (PACTR202003545370309).

### Sample size and sampling

The sample sizes were calculated by using Gpower 3.0. Sample size calculations were conducted for serum ferritin, serum folate, and blood hemoglobin concentration and the estimate that gave the largest sample size estimate (i.e. hemoglobin concentration) was retained. The power calculations assumed a power of 80%, an effect size of 0.3 and 20% non-response rate, which yielded a total sample size of 226 (n = 113/group).

In the four selected villages, a list of adolescent girls who were eligible for inclusion in the study was established with the help of the health extension worker. From this list, 226 adolescent girls were selected randomly through randomly generated numbers. Then, these adolescent girls (226 in total) were randomly assigned to the intervention group (G1 = 113) and the control group (G2 = 113).

Apparently healthy adolescent girls aged 10–19 years and willing to participate in the study were recruited from four villages from Wolaita and Hadiya zones. Adolescents that smoke or drink and with a known infectious disease like HIV/AIDS, Tuberculosis, and malaria (based on blood smear) were excluded. The initial screening was supported by physicians and laboratory tests were conducted at the Wolaita Sodo University referral hospital. Adolescents with hemoglobin < 12 g/dl (anemic) and > 17 g/dl (with increased risk of excessive intake) were excluded from the study.

### Anthropometric measurements

Anthropometric measures such as height, weight, and mid-upper arm circumference were measured at baseline and end line. Weight was measured to the nearest 100 g using a pre-calibrated digital scale (SECA), wearing light closing and without shoes. Height was measured in a standing position to the nearest 0.1 cm using a vertical board with a detachable sliding headpiece. Mid-upper arm circumference (MUAC) was measured using a standard MUAC tape on the upper left arm, after locating the midpoint for measurement between the end of the shoulder (acromion) and the tip of the elbow (olecranon).

### Biological/laboratory sample collection and analysis

An experienced phlebotomist drew fasting venous blood (~ 5 ml), from which drops were used to determine hemoglobin concentration in-field using a portable Hemocue photometer (Hb 301 + system). The remaining blood was allowed to settle at the data collection site (nearby health post) for 45 min before centrifugation to separate the serum. The serum was stored at − 20 °C overnight and was transferred to Wolaita Sodo University referral hospital, where it was stored at − 70 °C until further analyses. Samples were then transported on dry ice to the Ethiopian Public Health Institute (EPHI), where serum ferritin was analyzed using a fully automated clinical analyzer electrochemo-iluminescence immunoassay (ECLIA, Elecsys 2010 analyzer Cobas e 411; Roche Diagnostics GmbH, Mannheim, Germany); C-reactive protein (CRP) and Folate were determined by Immune turbid metric methods with a clinical chemistry analyzer (Cobas 6000 system; Roche Diagnostic GmbH). Hemoglobin concentrations were adjusted for altitude by using the following formula:

$${\text{Hb adjustment }} = - \;0.0{32 } \times \left( {{\text{altitude }} + \, 0:00{328}0} \right) \, + \, 0.0{2 } \times \left( {{\text{altitude }} + \, 0:00{328}0} \right)^{2}.$$

The Hb adjustment values obtained from the above calculation was subtracted from the measured hemoglobin concentration of each individual adolescent. Subjects with Hb values < 12 g/dl were considered anemic. CRP values > 5 µg/l were considered as elevated. Serum ferritin < 15.0 μg/l was considered as depleted iron stores. Serum folate between 3 and 5.9 ng/ml indicated marginal deficiency, whereas < 3 ng/ml is considered as deficient.

### Data quality assurance

The questionnaires were translated into the local languages: *Wolaitegna* and *Hadiya* and were back-translated to English to ensure the accuracy of the translation. Experienced data collectors, lab-technicians, and supervisors were recruited and trained for 4 days. The questionnaires were pre-tested and necessary changes were made prior to the actual surveys. The lead author and supervisors spot-checked the data collection and reviewed the questionnaires for completeness. Standardization of anthropometric measurements was taken. Anthropometric measurement was standardized by intensive training till the between and within measurement errors were reduced to an acceptable range. Acceptable technical error of measurement for weight was less than 0.1 kg, height was less than 0.5 cm and MUAC was less than 2 mm during standardization.

### Statistical analysis

Primary outcomes were analyzed based on an intention-to-treat principle. Socio-demographic characteristics were summarized as mean ± SD (or median and range) or frequency (percentage).

A comparison of means between the two arms was analyzed using a t-test. A multiple linear regression model was used to examine the impact of weekly IFA supplementation on hemoglobin concentration, serum ferritin, and serum folate, by running separate models adjusting for confounding factors like age, family size, weight, and baseline hemoglobin, serum ferritin, and serum folate concentrations. Multicollinearity was checked by using *variance inflation* factor (*VIF*), with a VIF values < 10 reflecting that mulitcollinearity is not a serious issue. The goodness of fit of the model was also checked.

All hypothesis tests were two-sided with a P-value < 0.05 considered as statistically significant. EP-data version 4.4.2 was used for data entry and SPSS version 21.0 was for analysis.

## Data Availability

All relevant data are within the paper and its supporting information file.
